# RUNX1-PDIA5 Axis Promotes Malignant Progression of Glioblastoma by Regulating CCAR1 Protein Expression

**DOI:** 10.7150/ijbs.92595

**Published:** 2024-08-12

**Authors:** Qiankun Ji, Zewei Tu, Junzhe Liu, Zhihong Zhou, Fengze Li, Xingen Zhu, Kai Huang

**Affiliations:** 1 Department of Neurosurgery, The Second Affiliated Hospital of Nanchang University, Nanchang, Jiangxi 330006, P. R. China.; 2 Jiangxi Key Laboratory of Neurological Tumors and Cerebrovascular Diseases, Nanchang, Jiangxi 330006, P. R. China.; 3 Institute of Neuroscience, Nanchang University, Nanchang, Jiangxi 330006, P. R. China.; 4 JXHC Key Laboratory of Neurological Medicine, Nanchang, Jiangxi 330006, P. R. China.; 5 Department of Neurosurgery, Zhoukou Central Hospital, Zhoukou, Henan 466000, P. R. China.; 6 Queen Mary School, Nanchang University, Nanchang, Jiangxi 330006, P. R. China.

**Keywords:** PDIA5, glioblastoma multiforme, malignant function, CCAR1, RUNX1

## Abstract

PDIA5 is responsible for modification of disulfide bonds of proteins. However, its impact on the malignant progression of glioblastoma multiforme (GBM) remains unknown. We analyzed the expression and prognostic significance of PDIA5 in cohorts of GBM and clinical samples. The PDIA5 protein was significantly overexpressed in GBM tissues, and higher expression of PDIA5 was statistically associated with a worse prognosis in patients with GBM. Transcriptional data from PDIA5 knockdown GBM cells revealed that downstream regulatory genes of PDIA5 were enriched in malignant regulatory pathways and PDIA5 enhanced the proliferative and invasive abilities of GBM cells. By constructing a PDIA5 CXXC motif mutant plasmid, we found CCAR1 was the vital downstream factor of PDIA5 in regulating GBM malignancy *in vitro* and *in vivo*. Additionally, RUNX1 bound to the promoter region of PDIA5 and regulated gene transcription, leading to activation of the PDIA5/CCAR1 regulatory axis in GBM. The RUNX1/PDIA5/CCAR1 axis significantly influenced the malignant behavior of GBM cells. In conclusion, this study comprehensively elucidates the crucial role of PDIA5 in the malignant progression of GBM. Downregulating PDIA5 can mitigate the malignant biological behavior of GBM both *in vitro* and *in vivo*, potentially improving the efficacy of treatment for clinical patients with GBM.

## Introduction

Glioblastoma multiforme (GBM) is the most lethal malignant intracranial tumor worldwide, accounting for approximately 57.3% of intracranial tumors. Despite decades of significant efforts to develop new therapy for GBM, the prognosis of patients has not improved significantly, with a median survival period of approximately 15 months and a 5-year survival rate of approximately 7.2% 1,2. The current main treatment strategy involves achieving maximum safe tumor resection, followed by postoperative radiotherapy combined with alkylating agent temozolomide chemotherapy 3,4. Unfortunately, complete resection of GBM is almost impossible due to its diffuse infiltration into adjacent brain tissues. Therefore, there is an urgent need for more effective and feasible treatment options for patients with GBM. A comprehensive understanding of the molecular mechanisms underlying the occurrence and progression of GBM will aid in the development of more effective targeted therapeutic drugs 5.

The protein disulfide isomerase (PDI) family comprises key proteins that regulate the spatial structure of proteins in the endoplasmic reticulum (ER) by controlling the formation and hydrolysis of disulfide bonds 6,7. Due to their crucial role in modulating the spatial structure of numerous proteins, PDIs are involved in various cellular biological activities essential for maintaining intracellular environmental homeostasis 8. Increasing research indicates that multiple members of the PDI family are involved in the malignant progression of cancer and therapeutic efficacy 9.

The PDIA5 gene, also known as PDIR, is located on chromosome 3q21.1 and encodes a protein consisting of 519 amino acids, which can be divided into 4 domains based on functional and structural characteristics, including an N-terminal ER recognition domain and three catalytic thioredoxin (TRX) domains (Cys-X-X-Cys) 10,11. The TRX domain binds to chaperone and calcium network proteins 12,13, the N-terminal cysteine is exposed on the protein surface, making the thiol group accessible for redox reactions, whereas the C-terminal cysteine has limited exposure. PDIA5 is highly expressed in most tumor cells and is located primarily in the lumen of the ER, where it catalyzes protein folding and thiol-disulfide bond exchange 14.

High levels of PDIA5 expression in macrophages can significantly affect tumor invasion within the immune microenvironment. PDIA5 is also involved in several tumor-related regulatory pathways to influence tumor progression, such as the PI3K/AKT, RTK signaling, and androgen receptor signaling pathways 15-17. Furthermore, by interacting with RNASET2, PDIA5 can be inactivated, reducing the ability of RNASET2 to produce uracil, and thus improving the cell's ability to capture exogenous uracil and enhancing cancer cell sensitivity to 5-fluorouracil (FU) 18. Furthermore, PDIA5 can promote ATF6 disulfide bond rearrangement of ATF6α under ER stress, maintaining its active conformation, and conferring chemotherapy resistance to cancer cells, thus affecting the progression of patients with glioma 19,20.

In this study, we found that PDIA5 is highly expressed in a variety of tumor tissues and is closely associated with a poor prognosis, particularly in GBM. Through RNA-seq analysis, LC-MS evaluation, and *in vitro* and *in vivo* experiments, we demonstrated that knocking down PDIA5 expression significantly impaired the malignant phenotypes of GBM via the PDIA5/CCAR1 signaling axis. Furthermore, we found that the expression of PDIA5 in GBM is transcriptionally regulated by RUNX1. Our results identify a new therapeutic target for patients with GBM and reveal the underlying mechanisms involved. Furthermore, our study suggests that targeting the RUNX1/PDIA5/CCAR1 signaling axis can interfere with the growth and progression of intracranial gliomas.

## Methods and Materials

### Public database data

This study included the RNA-sequencing data of four independent GBM cohorts, including TCGA (https://portal.gdc.cancer.gov/, n=144) 21, Rembrandt (http://gliovis.bioinfo.cnio.es/, n=179), Murat (http://gliovis.bioinfo.cnio.es/, n=80) 22, and Gravendeel (http://gliovis.bioinfo.cnio.es/, n=155) 23. Sequencing data was downloaded and matched with clinical data. The single cell sequencing data was downloaded from the GEO website (GSE182109) 24. Normal human RNA-seq data were obtained from the Genotype-Tissue Expression (GTEx) Portal (https://gtexportal.org/home/). Analysis combining TCGA data and GTEx data was performed on the Sangerbox platform (version 3.0; http://sangerbox.com/), which is a comprehensive and interaction-friendly clinical bioinformatics analysis platform 25.

### Acquisition of clinical samples

Tumor and adjacent paired tissue samples were obtained at the Neurosurgery Department of the Second Affiliated Hospital of Nanchang University from 2019 to 2022. The excision and preservation of the samples adhered to laboratory standards and strictly followed the Declaration of Helsinki guidelines. All patients who participated in this study signed informed consent forms and the study has been approved by the Ethics Committee of the Second Affiliated Hospital of Nanchang University.

### Reagents and antibodies

The antibodies used in this study were as follows: anti-GAPDH (Proteintech, Cat. No.10494-1-AP), anti-Tubulin (Proteintech, Cat. No. 11224-1-AP), anti-PDIA5 (Proteintech, Cat. No. 15545-1-AP), anti-Flag (Proteintech, Cat. No. 20543-1-AP), anti-HA (Proteintech, Cat. No. #66006-2-Ig), anti-GST (Cell Signaling Technology, Cat. No. 2622); anti-CCAR1 (ThermoFisher, Cat. No. PA5-78532), anti-N-cadherin (Proteintech, Cat. No. 22018-1-AP), anti-E-cadherin (Proteintech, Cat. No. 20874-1-AP), anti-MMP9 (Proteintech, Cat. No. 27306-1-AP), anti-MMP2 (Proteintech, Cat. No. 10373-2-AP), anti-pAKT (Proteintech, Cat. No. 66444-1-Ig), anti-PCNA (Proteintech, Cat. No. 10205-2-AP), anti-RUNX1 (Proteintech, Cat. No. 25315-1-AP). The experimental reagents used are as follows: RIPA lysis (Beyotime, Cat. No. P0013C), PMSF (Beyotime, Cat. No.# ST506), 4% paraformaldehyde (Beyotime, Cat. No. P0099), crystal violet solution (Solarbio Cat. No. G1062).

### Cell line acquisition and cell culture

The GBM cell lines used in this study, including U87MG, T98G, U118, LN229, and U251 MG, were purchased from the American Type Culture Collection (ATCC, USA), and the normal human astrocyte cell lines (NHA) and HEK293T cells were purchased from Shanghai cell bank. All cell lines described above were cultured with DMEM medium except for U87, which was cultured in 89%MEM/DMEM medium (Gibco, Cat. No.# 11380037 and 11960044) +10% FBS medium (Excell, Cat. No.# FCS500) +1% Pen-Strep (Solarbio Cat. No. P1400). Cells were placed in a cell incubator at 37°C, 5% CO2 and 100% humidity for cell culture, and all cells were treated when cell confluence reached approximately 80% (the logarithmic phase of cell growth).

### Western blotting

For clinical GBM tumor tissues, RIPA lysis buffer was used to extract total protein after cleaning the blood on the surface of the sample. After the cell protein suspension was separated, the BCA method was used to determine the concentration of the protein solution. The different molecular weights of the total proteins were separated by SDS-PAGE gels and the proteins were transferred to the PVDF membrane through the membrane transfer operation. The PVDF membrane was then sealed in 10% skim milk for no less than 2 h, followed by overnight incubation in primary antibody at the recommended dilution at 4°C. On the second day we incubated the bands at room temperature with secondary antibody for 3 h and finally incubated in ECL color development solution for band development in an exposure instrument. The images were saved and semiquantitative protein analysis was performed in ImageJ software. Note that the strips should be rinsed with TBST solution three times for 30 min each time before switching between different incubation fluids during the strip incubation process.

### Immunohistochemical staining

After fixing the GBM tumor tissues in paraformaldehyde solution, the tissues were sliced, and the pathological diagnosis was made by two pathologists with many years of clinical experience. After dewaxing, dehydration, peroxidase removal, antigen repair, primary and secondary antibody incubation, DAB and nuclear staining were performed. After the staining is complete, photographs are taken under the microscope and analyzed in the IHC Profiler package in ImageJ software. The IHC score is calculated using the following formula according to our previous study 26:



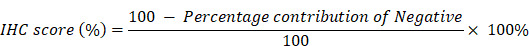



### Lentivirus construction and infection

The shCtrl, shPDIA5-1, shPDIA5-2, shCCAR1-1, shCCAR1-2, OE-Ctrl, OE-PDIA5, OE-CCAR1, and OE-RUNX1 lentiviruses were synthesized by Tianjin Shweis Biotechnology Co. LTD and U6-MCS-CMV-zsGreen-PGK-Puromycin plasmid vector was used to construct shCtrl, shPDIA5-1, shPDIA5-2, shCCAR1-1, and shCCAR1-2 plasmids, Ubi-MCS-SV40-EGFP-IRES-puromycin plasmid vector was used to construct OE-Ctrl, OE-PDIA5, OE-CCAR1, and OE-RUNX1 plasmids. Lentivirus infection was conducted according to the manufacturer's instructions. The shRNA sequence used in this study were: shPDIA5-1: 5'-CCACACTGTAAGAAGGTCATT-3'; shPDIA5-2: 5'-GCTCCTGAAGAAGGAAGAGAA-3'; shCCAR1-1: 5'-ATTGGTTGAAGCTACTTATAA-3'; shCCAR1-2: 5'-GCCCTAGTATGGAAGATTTAT-3'. All the constructed plasmids were lentiviral plasmids, which can form lentiviral particles in HEK293T cells with the help of lentiviral packaging plasmids (including pMD2.0 and psPAX2 plasmids, pMD2.0/psPAX2/lentiviral plasmids in the ratio of 1:1.5:2) for the construction of stable transfection cell lines. In this study, plasmid transfection was performed using Lipo3000 transfection reagent (Invitrogen, Cat. No. L3000015) and polybrene lentiviral-promoting transfection reagent (Sigma, Cat. No. TR-1003-G).

### RNA sequencing of cell sample

The shPDIA5 lentivirus was transfected into U251MG to construct a stable PDIA5 knockdown cell line and the corresponding negative control cell line was transfected at the same time. The PDIA5 protein knockdown efficiency was detected by western blotting. Next, the total mRNA of the two groups of cells was collected according to the RNA extraction kit, and the RNA sequencing was completed with the help of GENEWIZ. After initial quality control, the sequencing data was converted to reads per kilobase per million mapped reads (RPKM) for subsequent data analysis. Differentially expressed genes (DEG) analysis was performed using the package 'limma' (version 3.60.3) 27 to obtain DEG between the PDIA5 knockout group and the control group, and KEGG and GO enrichment analysis was performed through the package R package “clusterProfiler” (Version 4.12.0) 28.

### Wound healing assay

PDIA5/CCAR1-knockdown/overexpression and negative control cells were seeded in 6-well plates, respectively. When the cells reached 80% cell convergence, we used the tips of 200 μL pipette to create artificial scratches. After cleaning the cell fragments with PBS, the cells were cultured by replacing the low serum medium and the cells were photographed at 0 and 24 h, respectively. The scratch area was calculated using ImageJ software and Graphpad was used for statistical analysis.

### Transwell invasion experiment

GBM cells with different expression of PDIA5 or CCAR1 were seeded separately in Transwell chambers with 300 μL matrix gel in advance, and 200 μL cell suspension was added to each well with a density of 10×10^5^ cells /mL in non-serum medium. Next, 700 μL culture medium with 20% FBS was added to the lower chamber. Cells were observed for 24-48 h when the number of cells on the lower surface of the Transwell chamber was appropriate, and the lower cells of the chamber were fixed with 4% paraformaldehyde first, and the upper cells were gently wiped with a cotton swab. Cells were stained with 0.2% crystal violet solution for 30 minutes, after washing with PBS and then air-dried at room temperature. Stained cells were photographed under a microscope and ImageJ software was applied to count the number of migrated cells.

### The CCK-8 experiment

The two groups of GBM cells were divided into 96-well plates with 200 μL medium (10% FBS) and 1000 cells added to each well. After stable cell attachments, 10 μL CCK-8 solution was added to each well and incubated at 37°C for 1 h. The OD value was detected by the microplate reader at 450 nm wavelength every 24 h.

### Monoclonal formation experiment

First, 2 mL of DMEM medium and 800 GBM cells were added to each well of 6-well plates, respectively, and cultured for 2 weeks, during which cell growth was closely observed. When the cells grew to the appropriate density, we fixed the cells with 4% paraformaldehyde followed by cell staining with 0.1% crystal violet solution. Cell colonies counts are calculated using ImageJ software.

### EdU assay

GBM cells were seeded with or without PDIA5 or CCAR1 knockdown in 6-well plates on day 1. For staining, the cells were incubated in a cell incubator for 2 h with the addition of 20 μM EdU working solution in each well. The cells were then fixed and permeated in PBS solutions of 4% paraformaldehyde and 0.3% Triton X-100, respectively, followed by 0.5 mL of Click reaction solution configured according to the instructions in each well. Finally, the nuclei of Hoechst 33342 pairs were stained. Photographic records were taken under a fluorescence microscope.

### Immunoprecipitation and protein mass spectrometry

Flag-mut-PDIA5 expressing lentivirus was constructed and stably transfected into U251MG cells in the first step. Total protein of cells was extracted when the cells grew to an appropriate density. Anti-Flag antibody (Proteintech, Cat. No.# 20543-1-AP) was used to pull down proteins of interest through antigen antibody binding. The harvested protein samples were sent to the company for liquid chromatography-tandem mass spectrometry (LC-MS) to determine the protein bound with the mutant-PDIA5 protein.

### Immunofluorescence co-localization

U87MG and U251MG GBM cells were seeded in a 24-well plate. When the cells grew to the appropriate density, we fixed the cells with 4% paraformaldehyde solution and then the cells were treated with 0.3% Tritonx-100. Then, the steps of sealing, antibody incubation, corresponding fluorescent secondary antibody incubation, and DAPI nuclear staining were performed in turn, and the photos were obtained under a confocal laser microscope.

### Protein co-immunoprecipitation

Using U87MG and U251MG cells that stably overexpress Flag-mut-PDIA5 and HA-CCAR1, cell lysate (CST) was used to obtain cell lysate products and retain part of the proteins as the input group. Rabbit IgG (Cell signaling technology, Cat. No: 2729). To remove nonspecific binding proteins, samples were divided into two parts and incubated with Flag antibody and rabbit IgG antibody, respectively, on a rotating shaker at 4°C for 6 h. After washing, the samples were heated in a metal bath at 100°C for 8 minutes using a 2x loading buffer solution of 20 μL. After centrifugation, the supernatant was taken as a protein sample for subsequent western blotting. The target protein was incubated with HA antibody.

### GST pull-down assay

Human PDIA5 and CCAR1 genes were cloned into pET-28a vectors, tagged with GST and HA, respectively, to construct the GST-PDIA5 and HA-CCAR1 fusion proteins. The recombinant plasmids were then transformed into *E. coli* BL21 (DE3) cells. Cultures were grown to an OD 600 of 0.6, and protein expression was induced with IPTG (Beyotime, China) for 16 h. Bacterial cells were lysed using a buffer containing PMSF. Lysates containing GST or GST-PDIA5 were incubated with glutathione agarose beads at 4°C for 2 h. After washing the beads five times, the HA-CCAR1 lysate was added and incubated for 6 h at 4°C. The beads were washed five additional times and the bound complexes were eluted with glutathione elution buffer. The eluates were mixed with sample buffer and boiled at 100°C for 5 minutes for western blotting analysis.

### Xenograft tumor implantation model in nude mice

Fifty-four-week-old male BALB/c nude mice (GemPharmatech, Nanjing, China) were applied as the recipient of intracranial GBM xenografts, and the U87MG cell line was selected for the animal tumor formation studies. We suspended the GBM cells with pre-cooled PBS solution and adjusted the cell concentration to 5×10^7^ cells/mL by cell counter. Each mouse was injected with 6 μL tumor cells, and the injection point was 2 mm outside and 1 mm behind the anterior fontanel 29. Tumor size was characterized by the intensity of fluorescence signal displayed in the IVIS system by intraperitoneal injection of potassium salt luciferin (PerkinElmer, Cat. No. 122799-5), and the weight, activity status, and tumor size of the mice were regularly monitored. The mice were raised in a Specific Pathogen-Free Animal House (SPF) and surgical procedures were applied under strict aseptic standards. When the mice showed severe weight loss of more than 30% or abnormal activity, the mice were euthanized by CO_2_ inhalation and brain tissues were completely removed and the samples were fixed in 4% paraformaldehyde at 4°C for storage. The animal experiment was approved by the Medical Ethics Committee of the Second Affiliated Hospital of Nanchang University.

### Prediction and validation of PDIA5 upstream transcription factors (ChIP-PCR)

The sequence of the promotor region of PDIA5 (2000 bp nucleic acid sequence upstream of the promoter) was obtained from the UCSC database, and JASPAR predicted that RUNX1 had a trusted binding site in the PDIA5 promoter region. We then tested whether RUNX1 could bind to the promoter region of PDIA5 using the ChIP assay. The ChIP assay was performed according to the standard protocol. The above prediction results show that RUNX1 has 5 high-confidence regions in the PDIA5 promoter region, and 5 pairs of ChIP-PCR primers were designed from the DNA sequences in these regions: Region1: F: 5'-TTGGATGGGCCTGTTGTGAATAGG-3' and R: 5'-ACAGCACAGTCAGAGGTTGGT-3'; Region2: F: 5'-AACTGACTGCATGCAAGAATG-3' and R: 5'-TAATGCCTGATGATCCGAGATAG-3'; Region3: F: 5'-TAGCACAGGGAATGGGTTCAA-3 and R: 5'-GGAGATGATGGTTCCACAACATT-3'; Region4: F: 5'-AGAGTTGTTTCTACCTTTTGGTTA-3' and R: 5'-GAAAACAATCTGGTGGTTCCTCA-3'; Region5: F: 5'-GAGGAACCACCAGATTGTTT-3 'and R: 5'-AGCCGGTAAATGGCAGAGTC-3'. DNA samples were amplified by PCR, and the products were subjected to DNA electrophoresis, and the PCR amplification content in different regions was compared to determine the binding region of the RUNX1 and PDIA5 promoter.

### Dual-luciferase reporter assay

Diverse PDIA5 promoter sequences (including full-length sequence, region 1 mutated sequences, and region 1 only sequence, respectively) were cloned into the firefly-luciferase reporter plasmid. Plasmids were transfected into U87 and U251 cells (including RUNX1 overexpression and control cells) in 96-well plates. After 48 h of plasmid transfections, U87 and U251 GBM cells were harvested, and luciferase activity was quantified using the dual-luciferase reporter assay kit (Cat. No #RG029M, Beyotime, Shanghai, China) according to the manufacturer's instructions.

### Statistical analysis

R software (version 4.0.1) and GraphPad 8.0 software were used for statistical analysis and data visualization. The Kaplan-Meier survival curve was used for survival analysis and the statistical method was the log-rank test. The differential expression between the two groups was analyzed using the Wilcox rank sum test. The t-test for unpaired comparison was used to analyze the difference between three repetitions of experimental data. The results of western blotting bands and immunohistochemical staining score were quantified by ImageJ software. A p-value <0.05 was considered statistically significant.

## Results

### PDIA5 was highly expressed in GBM and predicted a poor prognosis

**Figure 1:**
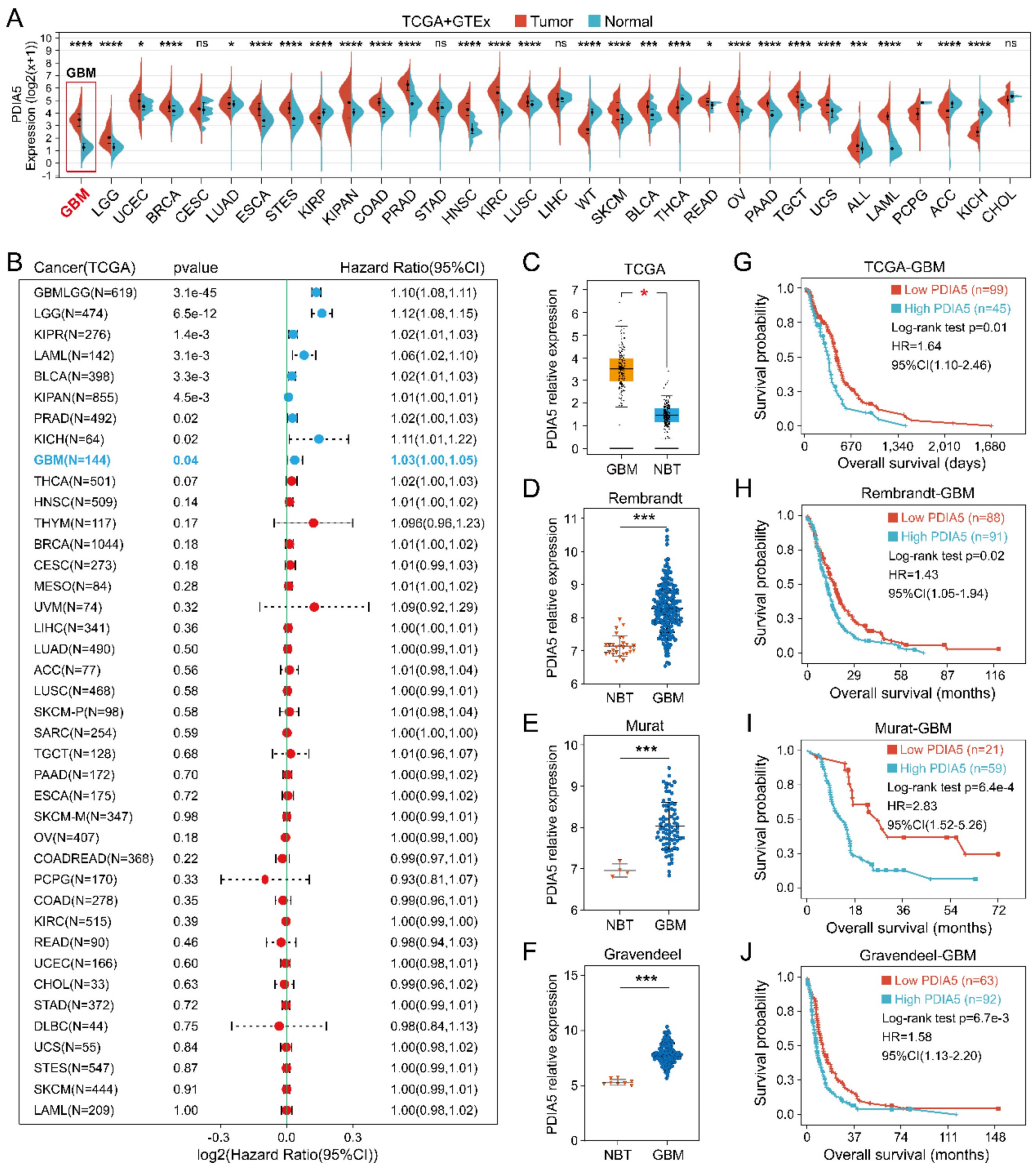
** (A)** The expression of PDIA5 mRNA was significantly higher in GBM tissues than in normal brain tissues. **(B)** PDIA5 mRNA has prognostic significance in GBM patients and is associated with poor prognosis. **(C-F)** Differential expression analysis of PDIA5 mRNA between GBM samples and normal brain tissues from the Gliovis website. **(D-F)** Differential expression analysis of PDIA5 mRNA in Rembrandt** (D)**, Murat **(E)**, and Gravendeel** (F)** datasets. **(G-J)** Analysis results from the TCGA-GBM **(G)**, Rembrandt **(H)**, Murat** (I)**, and Gravendeel **(J)** datasets showed higher expression of PDIA5 mRNA is associated with worse prognosis of GBM patients.

The PDIA5 transcriptome expression data in 33 tumor types were analyzed, including tumor sample data from TCGA and normal sample data from GTEx datasets. As shown in **Figure 1A,** the expression of PDIA5 mRNA was upregulated in most tumor tissues, especially GBM. The prognostic significance of PDIA5 mRNA was then evaluated in various cancers. The results indicated that PDIA5 mRNA has significant prognostic value in various tumor types, all associated with a poor patient prognosis, including GBM, LGG, KIRP, LAML, BLCA, KIPAN, PRAD, and KICH** (Figure 1B)**. Differential expression analysis and survival analysis were performed using different GBM datasets, validating these findings in the TCGA-GBM, Rembrandt, Murat, and Gravendeel GBM cohorts. PDIA5 consistently showed a significantly higher expression in GBM tissues **(Figure 1C-F)**, and patients with higher expression of PDIA5 had worse clinical outcomes **(Figure 1G-J)**. These results suggest that PDIA5 may play a crucial role in promoting cancer progression in patients with GBM.

### Validation of high expression of PDIA5 in clinical GBM tissues

**Figure 2:**
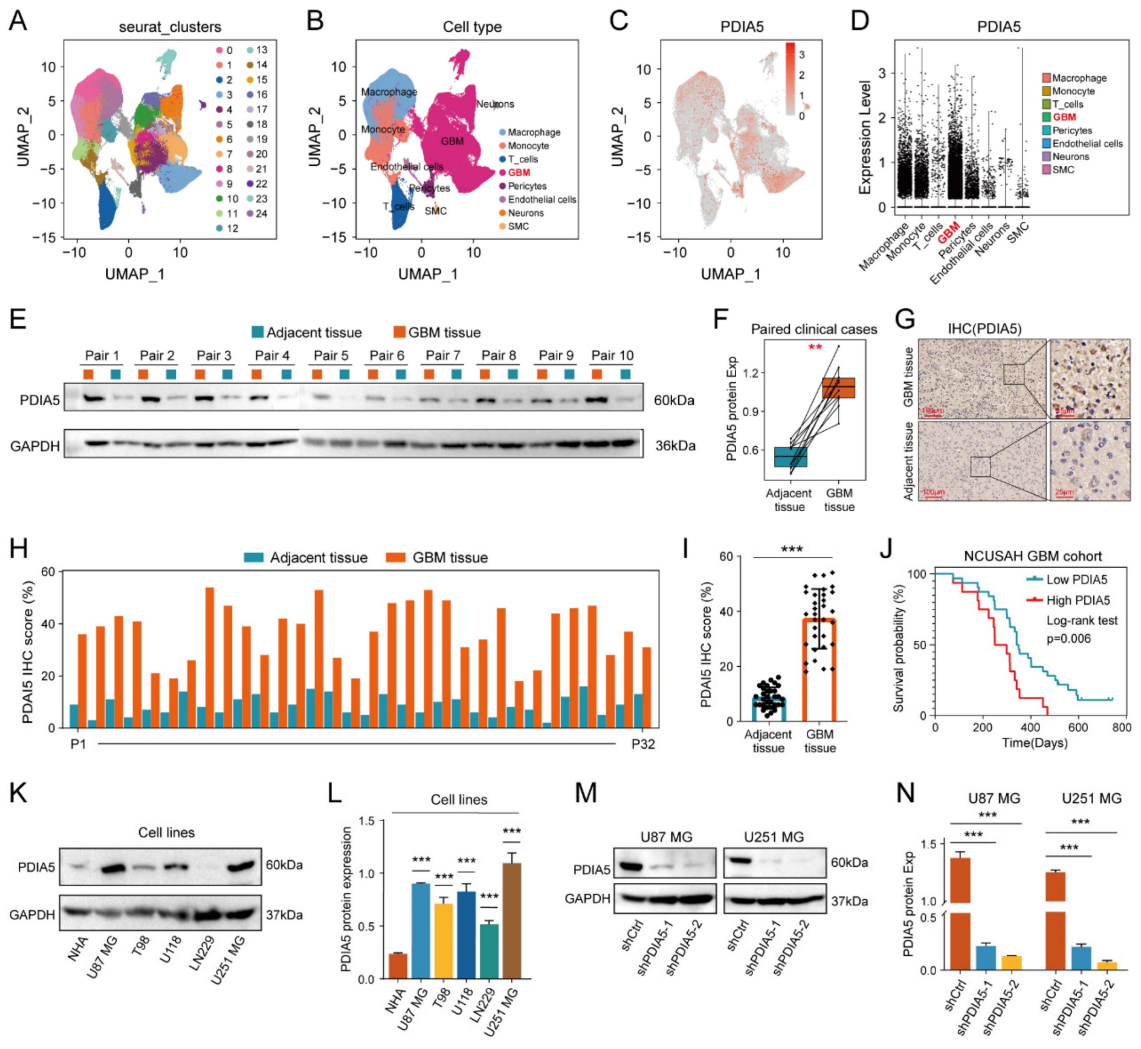
** (A)** Twenty-four cell clusters were obtained as a result of sequencing data processing. **(B)** The cell clusters were annotated according to the expression of the marker genes, resulting in 8 cell types. **(C)** PDIA5 mRNA is widely expressed among different cell types. **(D)** The expression of PDIA5 mRNA was higher in GBM cells. **(E-F)** Western blotting detected the differential expression of PDIA5 protein between GBM tissues and corresponding adjacent brain tissues. **(G)** Differential expression of PDIA5 protein in GBM tissues and corresponding adjacent brain tissues was detected by IHC. **(H)** Analysis of the differential expression of IHC scores between each GBM tissue pair and adjacent brain tissue. **(I)** Analysis of overall differential expression of IHC scores between GBM tissues and adjacent brain tissues. **(J)** Prognostic analysis of IHC score in GBM patients. **(K-L)** Western blotting detected the differential expression of PDIA5 protein between NHA cell lines and GBM cell lines. **(M-N)** Western blotting detected changes in expression of PDIA5 protein in U87MG and U251MG cells infected with lentivirus.

We explore the differences in PDIA5 expression between various types of types of GBM tissue cells by conducting single-cell sequencing data analysis, which included sequencing data from 13 patients with GBM and 18 patients with lower-grade glioma (LGG). PDIA5 was predominantly expressed in GBM cells and macrophages **(Figure 2A-D)**. Next, we surgically removed tissue samples from clinical patients, including GBM tissues and adjacent tissues, and western blotting results demonstrated that the expression of the PDIA5 protein was significantly higher in GBM tissues compared with adjacent brain tissues **(Figure 2E-F)**. Similarly, immunohistochemical staining of 32 pairs of tissue slices was performed and scored, confirming that PDIA5 was significantly overexpressed in GBM samples compared to adjacent tissues **(Figure 2G-I)**. Based on the histochemical scores of clinical samples and patient follow-up data, the clinical samples were divided into high- and low-PDIA5 expression groups. Kaplan-Meier survival analysis confirmed that patients with high expression of PDIA5 had a worse prognosis **(Figure 2J)**. Altogether, this evidence suggests that PDIA5 may play a key role in the progression of GBM. To explore the underlying mechanism by which PDIA5 may promote the malignant progression of GBM and its regulation in GBM, we conducted and found that U87MG and U251MG cells have the highest expressions of PDIA5 **(Figure 2K-L)**. We then transfected PDIA5 knockdown lentivirus into U87MG and U251MG cell lines and performed WB to determine the knockdown effect **(Figure 2M-N)**.

### RNA sequencing combined with enrichment analysis predicted the involvement of PDIA5 in glioma cells

**Figure 3:**
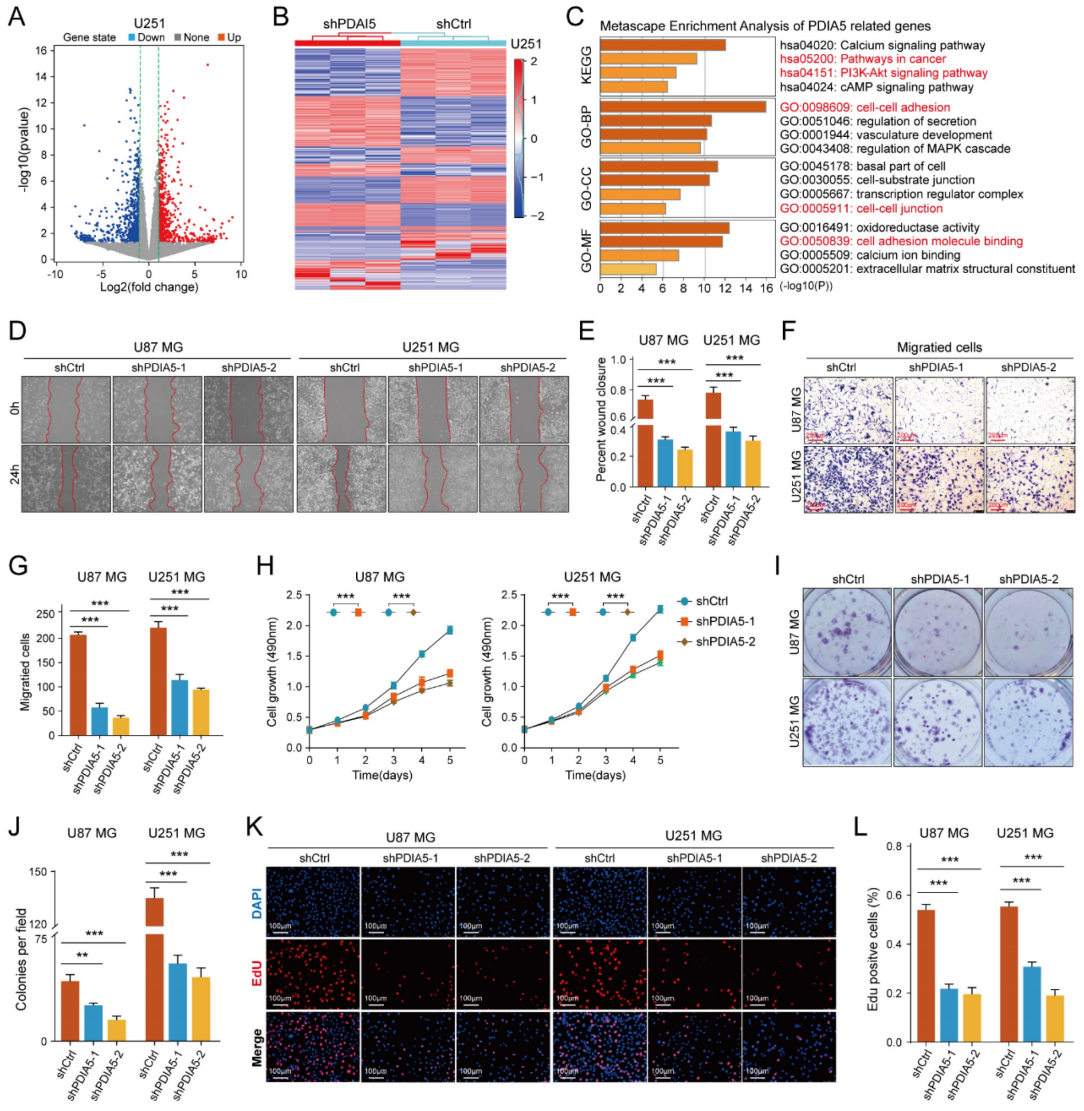
** (A)** Volcano plot identifying the differentially expressed genes (DEGs) between sh-Ctrl and sh-PDIA5 U251MG cell lines. **(B)** DEGs between sh-Ctrl and sh-PDIA5 U251MG cell line were visualized in this heatmap. **(C)** The Metascape enrichment analysis represents the gene ontology (GO) and KEGG pathway terms which these DEGs enriched.** (D-E)** Effects of PDIA5 knockdown on wound-healing ability of U87MG and U251MG cells as detected by wound-healing experiment. **(F-G)** Transwell invasion assay detect the effects of PDIA5 knockdown on invasion ability of U87MG and U251MG cells. CCK-8 assay **(H)**, monoclonal formation assay** (I-J),** and EdU assay **(K-L)** jointly verified the effects of PDIA5 knockdown on the proliferation ability of U87MG and U251MG cells.

To determine the cellular process regulating GBM malignancy potentially involving PDIA5, we used PDIA5 knock-down U251MG cells for RNA sequencing analysis. Using the "limma" package, we identified genes whose expression changed significantly after PDIA5 knockdown (p<0.05; FDR<0.05). These genes were then visualized using volcano plots and heatmaps **(Figure 3A-B)**. KEGG and GO enrichment analysis (including GO-BP, GO-CC, and GO-MF) revealed that PDIA5 expression was closely related to various cancer-related signaling pathways and cell functions, including the PI3K-Akt signaling pathway **(Figure 3C).** This suggested a connection between PDIA5 and tumor cell malignancy.

We then tested the malignant phenotypes between PDIA5 knockdown and negative control U87MG and U251MG GBM cells. Wound healing assays showed that the cell migration ability was significantly weakened with PDIA5 knock-down** (Figure 3D-E)**; Transwell invasion assays also indicated that the invasion ability of the GBM cells declined after PDIA5 inhibition **(Figure F-G).** Furthermore, the CCK-8 and EdU assays demonstrated that the proliferation ability of tumor cells was reduced after PDIA5 removal **(Figure 3I-L)**. All the results support our hypothesis that PDIA5 plays a crucial role in the malignant progression of GBM. Meanwhile, we also compared the invasive and proliferative abilities between PDIA5 overexpression GBM cells and negative control GBM cells. Consistent with previous data, we also found that overexpression of PDIA5 can significantly promote the invasion and proliferation of U87 and U251 GBM cells **(Supplementary Figure 5)**.

### IP-MS screening and validation of downstream substrates of PDIA5

**Figure 4:**
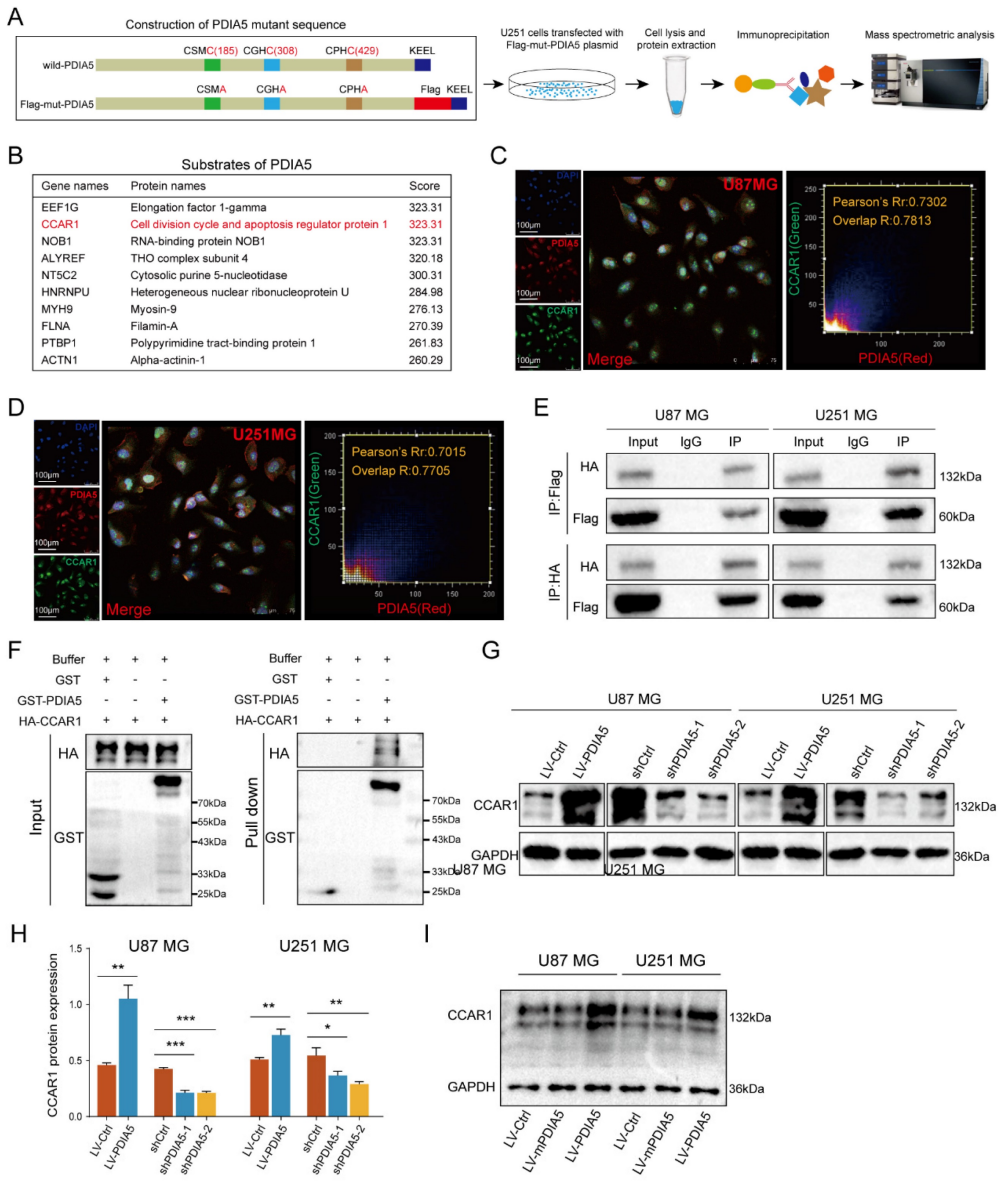
** (A)** Construction, transfection, protein extraction and mass spectrometry of PDIA5 mutant plasmid. **(B)** Mass spectrometry results are presented in the table. Immunofluorescence co-localization detection of PDIA5 and CCAR1 in **(C)** U87MG and **(D)** U251MG cells. **(E)** Co-IP assay confirmed the presence of mutual binding between PDIA5 and CCAR1 proteins. **(F)** GST-PDIA5 interacts with HA-CCAR1 in vitro as indicated by GST pull-down assay. **(G-H)** Western blotting verified that PDIA5 regulated CCAR1 protein expression. **(I)** The effects of wildtype PDIA5 and mutant PDIA5 overexpression on regulating CCAR1 protein expressions in U87 and U251 GBM cells.

To further explore how PDIA5 affects GBM cell malignancy, we designed an immunoprecipitation mass spectrometry (IP-MS) assay to identify downstream proteins that interact with PDIA5 in GBM cells. Based on previous reports, we designed and constructed a mutant plasmid at the active site of PDIA5, the mutant protein PDIA5 prolongs the binding time with substrate proteins 26,30,31. Lysates from U251MG GBM cells transfected with mPDIA5 were collected for immunoprecipitation and substrate proteins were harvested and then analyzed by liquid chromatography and tandem mass spectrometry detection **(Figure 4A)**. Considering that we have shown that PDIA5 can accelerate GBM cell proliferation and invasion, we focused on the top10 potential substrates of PDIA5 (**Figure 4B).** Among these, the cell division cycle and apoptosis regulator protein 1 (CCAR1) had the highest score. We speculated that PDIA5 facilitates GBM cell proliferation and invasion by regulating the maturation and correct folding of CCAR1. Next, we performed a subcellular immunofluorescence colocalization experiment using U87MG and U251MG cells to detect the cell localization of both PDIA5 and CCAR1. Both proteins were expressed in the cytoplasm and nucleus, showing a high degree of colocalization, indicating a possible interaction between PDIA5 and CCAR1 in GBM cells **(Figure 4C-D)**. To further verify a direct interaction between PDIA5 and CCAR1, we constructed an HA tag labeled CCAR1 (HA-CCAR1) and performed a bidirectional immunoprecipitation. The results confirmed a direct interaction between the two proteins **(Figure 4E)**. In addition, we also verified the interaction between CCAR1 and PDIA5 by GST pulldown assay in *E. coli* expression system** (Figure 4F)**, the results also support the direct interaction between PDIA5 and CCAR1. After proving the direct interaction between PDIA5 and CCAR1, we explored whether PDIA5 regulates the expression of CCAR1. We found that PDIA5 overexpression in U87MG and U251MG cells increased CCAR1 protein levels, while PDIA5 suppression decreased CCAR1 protein levels **(Figure 4G-H)**. Furthermore, we also compared the expression of the CCAR1 protein between GBM cells with overexpression of wild-type PDIA5 and mutated PDIA5 motifs of CXXC, and the results showed that the CCAR1 protein was up-regulated in LV-PDIA5 rather than GBM cells LV-mPDIA5** (Figure 4I)**. Thus, we conclude that PDIA5 improved the expression of the CCAR1 protein by regulating its correct folding and maturation.

### Knockdown of CCAR1 inhibited migration and invasion of GBM cells

**Figure 5:**
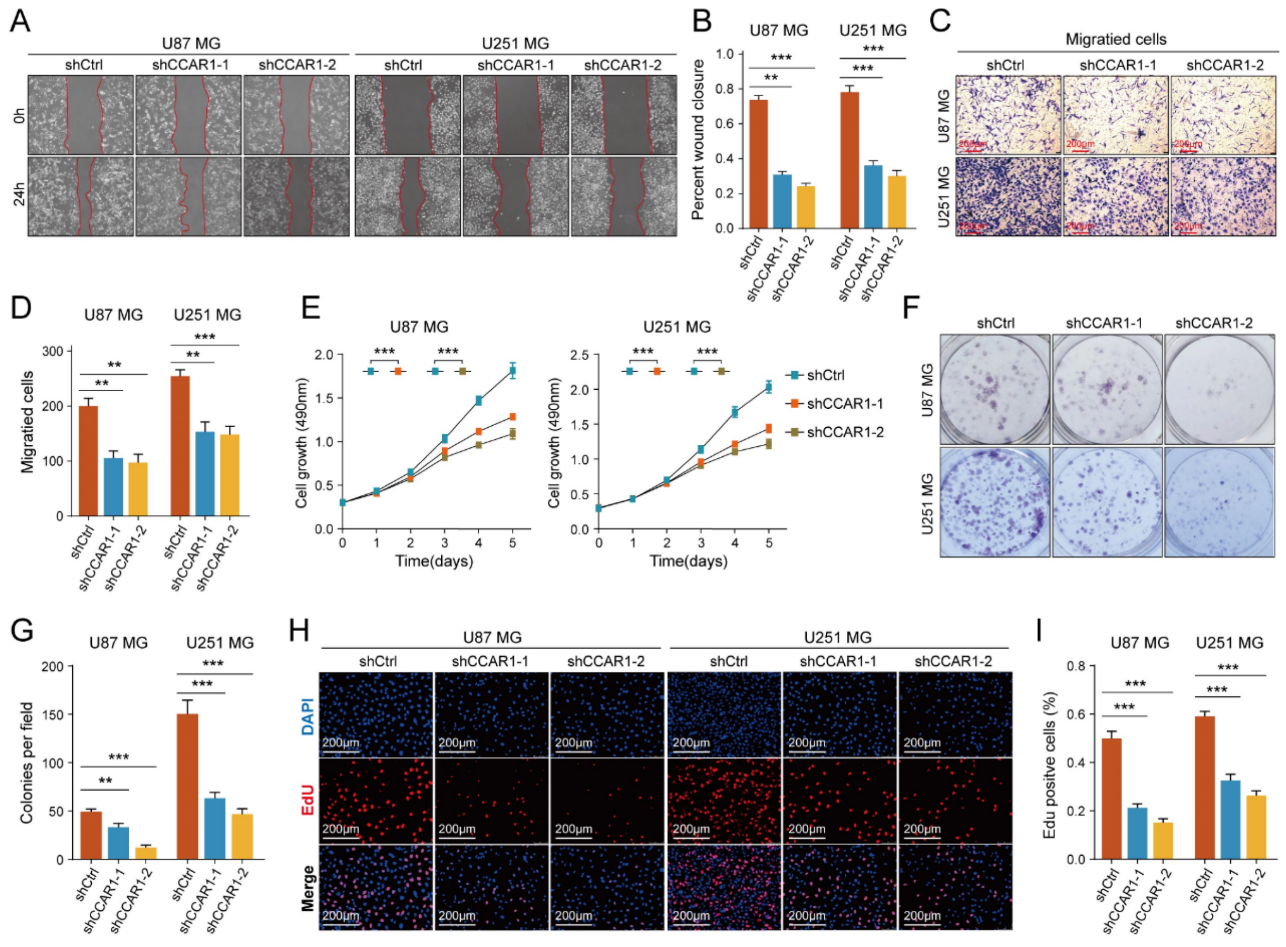
The effect of knockdown CCAR1 on wound-healing ability of **(A)** U87MG and **(B)** U251MG cells was detected by wound-healing experiment. The Transwell invasion assay was used to detect the effects of knocking down CCAR1 on invasion ability of **(C)** U87MG and **(D)** U251MG cells. **(E)** CCK-8 assay, **(F-G)** monoclonal formation assay and **(H, I)** EdU assays jointly verified the effects of CCAR1 knockdown on the proliferation ability of U87MG and U251MG cells.

The role of CCAR1 in tumor cell migration, invasion, and proliferation has been validated in various tumors, but its functional effects in GBM cells have not been studied. We designed two CCAR1 knockdown shRNAs and packaged lentivirus to transfect U87MG and U251MG cells. Stable CCAR1 knockdown GBM cell lines were then used to repeat the cell proliferation and invasion experiments mentioned above. The results of wound healing experiments showed that the migration abilities of U87MG and U251MG cells expressing sh-CCAR1s were significantly reduced compared with sh-Ctrl GBM cells **(Figure 5A-B)**. The Transwell invasion experiment demonstrated that the invasive abilities of CCAR1 knockdown U87MG and U251MG cells were also significantly weakened compared with negative control GBM cells** (Figure 5C, D)**. The CCK-8 assay showed that U87MG and U251MG cells grew slower after sh-CCAR1 lentivirus transfections **(Figure 5E)**. Consistently, monoclonal formation experiments also indicated that the clonal formation ability of U87MG and U251MG cells was significantly weakened when CCAR1 was blocked **(Figure 5F, G)**. Furthermore, the EdU results consistently support that inhibition of CCAR1 can slow GBM cell growth **(Figure 5H-J)**. Furthermore, we also compared the invasive and proliferative abilities between GBM cells overexpressing CCAR1 and negative control GBM cells. Consistent with previous data, we also found that overexpression of CCAR1 could significantly promote invasion and proliferation of U87 and U251 GBM cells **(Supplementary Figure 6)**.

### PDIA5 affected the invasion and proliferation ability of GBM cells through CCAR1

**Figure 6:**
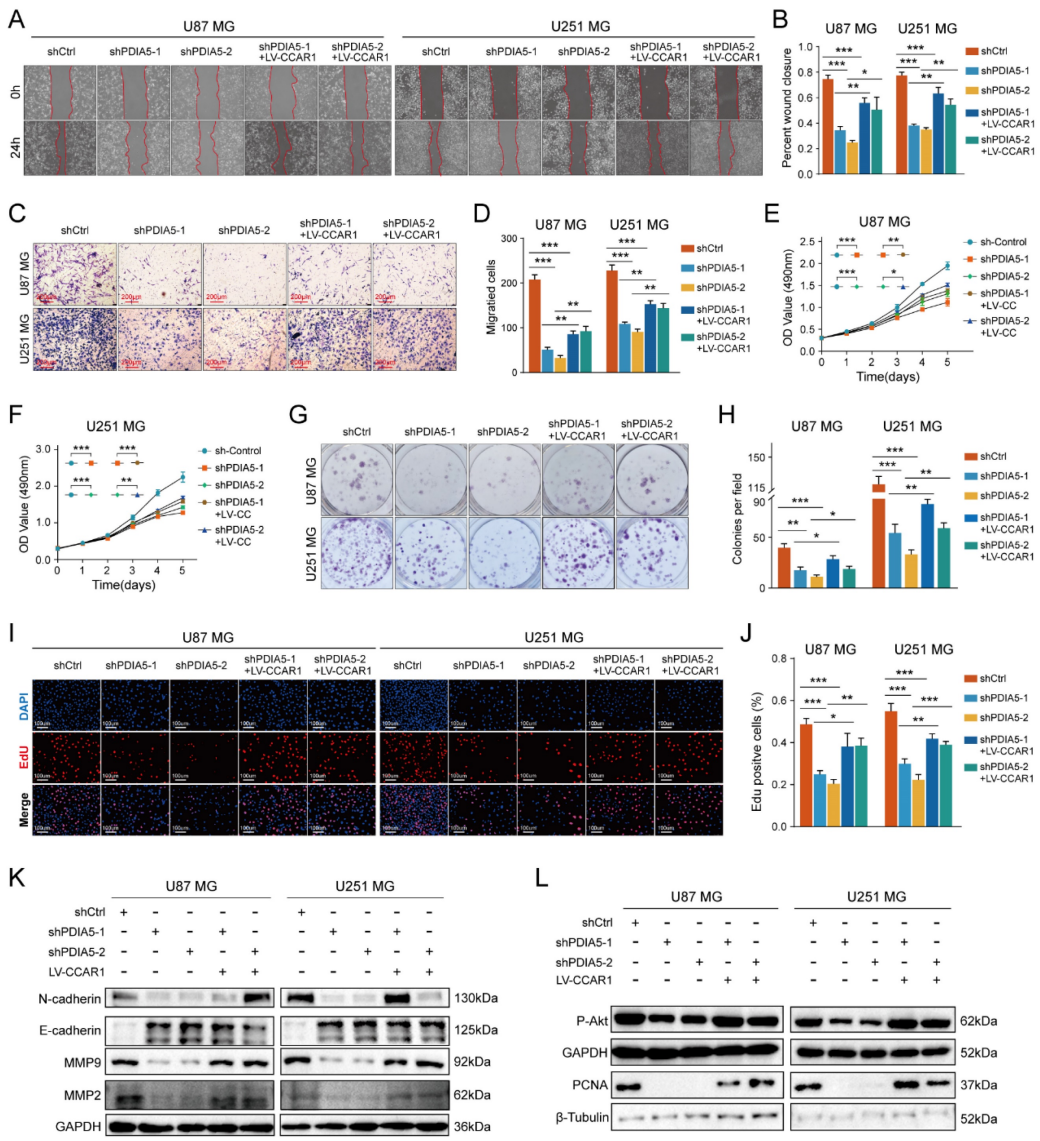
Wound healing recovery showed that overexpression of CCAR1 could partially rescue the effects of PDIA5 knockdown on the wound healing ability of **(A)** U87MG and **(B)** U251MG cells. Transwell invasion recovery experiment showed that overexpression of CCAR1 could partially rescue the effects of PDIA5 knockdown on invasion ability of **(C)** U87MG and **(D)** U251MG cells. The CCK-8 assay **(E-F)**, monoclonal formation assay **(G-H)**, and EdU assay **(I-J)** jointly verified that overexpression of CCAR1 could partially rescue the effects of PDIA5 knockdown on the proliferation ability of U87MG and U251MG cells. **(K)** Western blotting detected the protein expression of U87MG and U251MG cell migration and invasion marker genes in different groups. (L) Western blotting detected the protein expressions of cell proliferation marker genes in U87MG and U251MG cells in different groups.

To test our hypothesis that PDIA5 promotes GBM cell proliferation and invasion by regulation of CCAR1 protein expression, we constructed a lentivirus expressing CCAR1 to determine whether overexpression of CCAR1 could rescue GBM cell proliferation and invasion abilities after PDIA5 knockdown. The effect of CCAR1 overexpression was validated by WB assay **(Figure S3A-D)**. The wound healing assay and the Transwell invasion assay were used to verify whether CCAR1 overexpression could reverse the effects of PDIA5 knockdown on GBM cell migration and invasion. The migration ability of U87MG and U251MG cells with PDIA5 knockdown was significantly improved after overexpression of CCAR1 **(Figure 6A-B)**. Similarly, the invasion ability of U87MG and U251MG cells in shPDIA5-1+LV-CCAR1 and shPDIA5-2+LV-CCAR1 GBM cells was significantly improved compared to shPDIA5-1 and shPDIA5-2 GBM cells** (Figure 6C-D)**. These results suggest that overexpression of CCAR1 can partially salvage defects induced by PDIA5 knockdown in GBM cells. Next, we verified that PDIA5 accelerates the proliferation of GBM cells through CCAR1 by conducting rescue experiments. The results of the CCK-8 assay** (Figure 6E, F)**, monoclonal formation assay **(Figure 6G, H),** and the EdU staining** (Figure 6I, J)** all showed that CCAR1 overexpression could partially rescue the decreased proliferation of U87MG and U251MG cells caused by PDIA5 suppression. Western blotting was used to detect the expression of relevant functional proteins and revealed that their expression was consistent with the functional characterization of cells **(Figure 6K, L)**.

### PDIA5 influences the formation of glioblastoma *in vivo* through CCAR1

**Figure 7:**
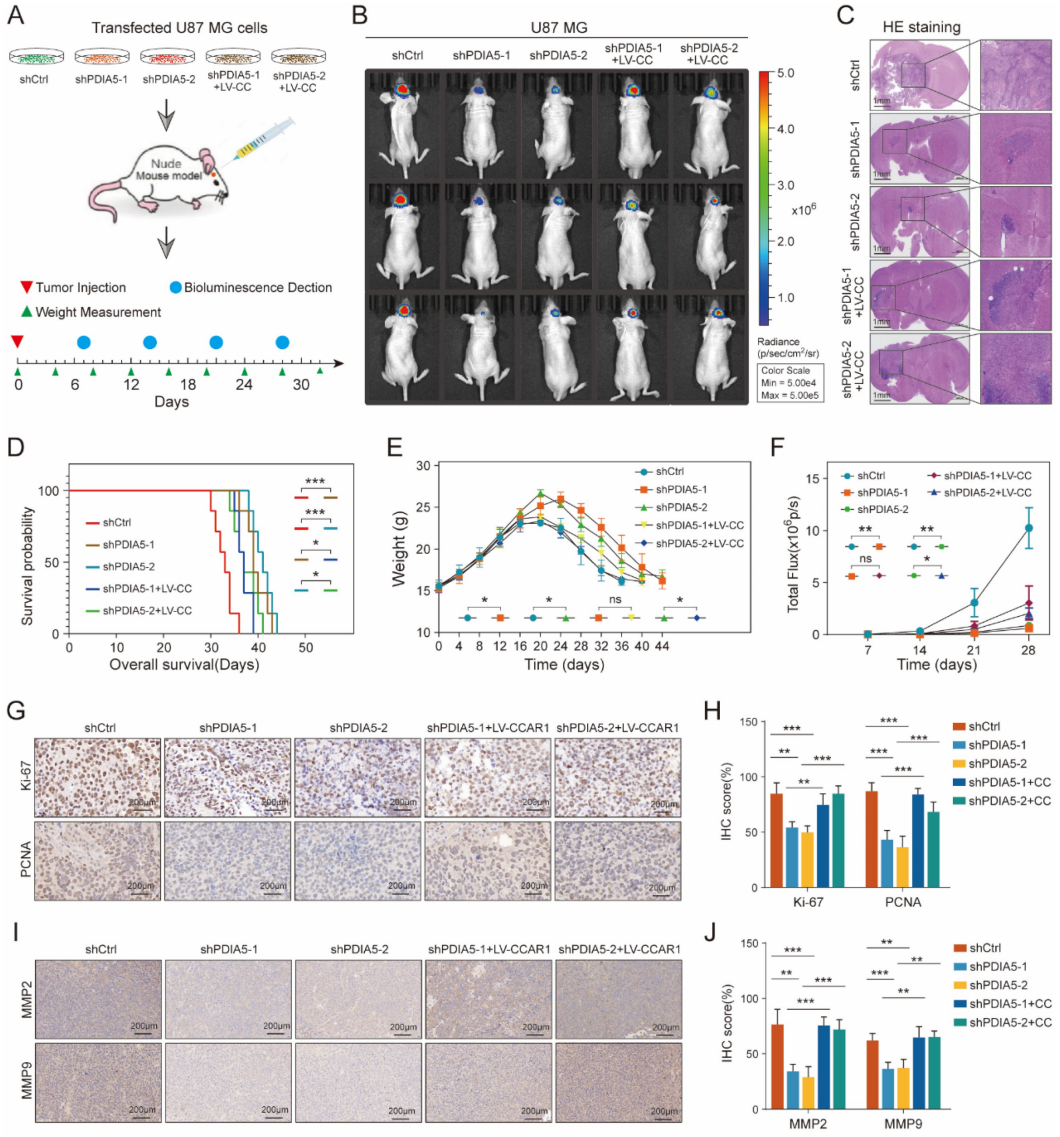
** (A)** Procedure for intracranial xenotransplantation of glioblastoma. **(B, C)** In vitro imaging and HE staining showed the tumor size and fluorescence intensity of shCtrl, shPDIA5-1, shPDIA5-2, shPDIA5-1+LV-CCAR1, and shPDIA5-2+LV-CCAR1 U87MG xenografts in nude mice. **(D)** The line chart shows the difference analysis of the overall survival time of nude mice between different groups. **(E)** Line plots showed changes and differences in the body weight of nude mice at different time points among different groups.** (F)** Line plots showed the trend and difference analysis of total fluorescence intensity in different groups. **(G)** Representative images of IHC staining of Ki67 and PCNA protein molecules.** (H)** Differential expression analysis of Ki67 and PCNA protein molecules by IHC staining.** (I)** Representative images of IHC staining of MMP2 and MMP9 proteins.** (J)** Quantification of MMP2 and MMP9 protein molecules by IHC staining.

To further validate whether PDIA5 influences glioblastoma malignancy through CCAR1 *in vivo*, we injected stable transfected U87 cell lines (shCtrl, shPDIA5-1, shPDIA5-2, shPDIA5-1+LV-CCAR1 and shPDIA5-2+LV-CCAR1) into the brain of nude mice using a stereotaxic instrument to form intracranial GBMs **(Figure 7A)**. During the breeding period, mice were regularly weighed and photographed using the IVIS system to observe the growth state of brain tumors, and survival time was counted and recorded in detail. The results showed that compared to the sh-Ctrl group, the size of the intracranial tumor of nude mice in the shPDIA5-1 and shPDIA5-2 groups was significantly reduced** (Figure 7B-C)**, the overall survival time was significantly extended **(Figure 7D)**, weight gain was more gradual and the decline was slower and gradual **(Figure 7E)**. Additionally, the overall fluorescence intensity was significantly lower** (Figure 7F)**. These effects of PDIA5 suppression was partially reversed by overexpression of CCAR1 *in vivo* **(Figure 7B-F)**. In addition, IHC staining was performed on tissue sections of intracranial transplanted GBMs in nude mice to determine changes in the proliferation ability of tumor cells primarily by targeting two cell cycle-related molecules, Ki67 and PCNA. We found significantly lower Ki67 and PCNA positive rates in the shPDIA5-1 and shPDIA5-2 groups compared with the shCtrl group. The positive rates of Ki67 and PCNA in shPDIA5-1+LV-CCAR1 and shPDIA5-2+LV-CCAR1 groups were higher than those in shPDIA5-1 and shPDIA5-2 groups, which means CCAR1 could rescue the effects of PDIA5 inhibition on cell proliferation **(Figure 7G, H)**. Consistent results with cell invasion markers MMP2 and MMP9 were observed. The removal of PDIA5 knockdown inhibited the expression of MMP2 and MMP9 *in vivo*, whereas the overexpression of CCAR1 rescued the expression of MMP2 and MMP9 in xenografts without PDIA5 expression **(Figure 7I, J)**. Overall, the results of IHC staining showed that PDIA5 could affect the proliferative and invasive characteristics of intracranial transplanted tumors in nude mice through CCAR1.

Summarizing the *in vivo* data, inhibition of PDIA5 postponed the progression of intracranial GBM in nude mice, but the effect could be rescued by overexpression of CCAR1. This indicates that PDIA5 promotes GBM progression by regulating CCAR1 expression *in vivo*.

### RUNX1 acted as a transcription regulator to regulate the transcription process of PDIA5

**Figure 8:**
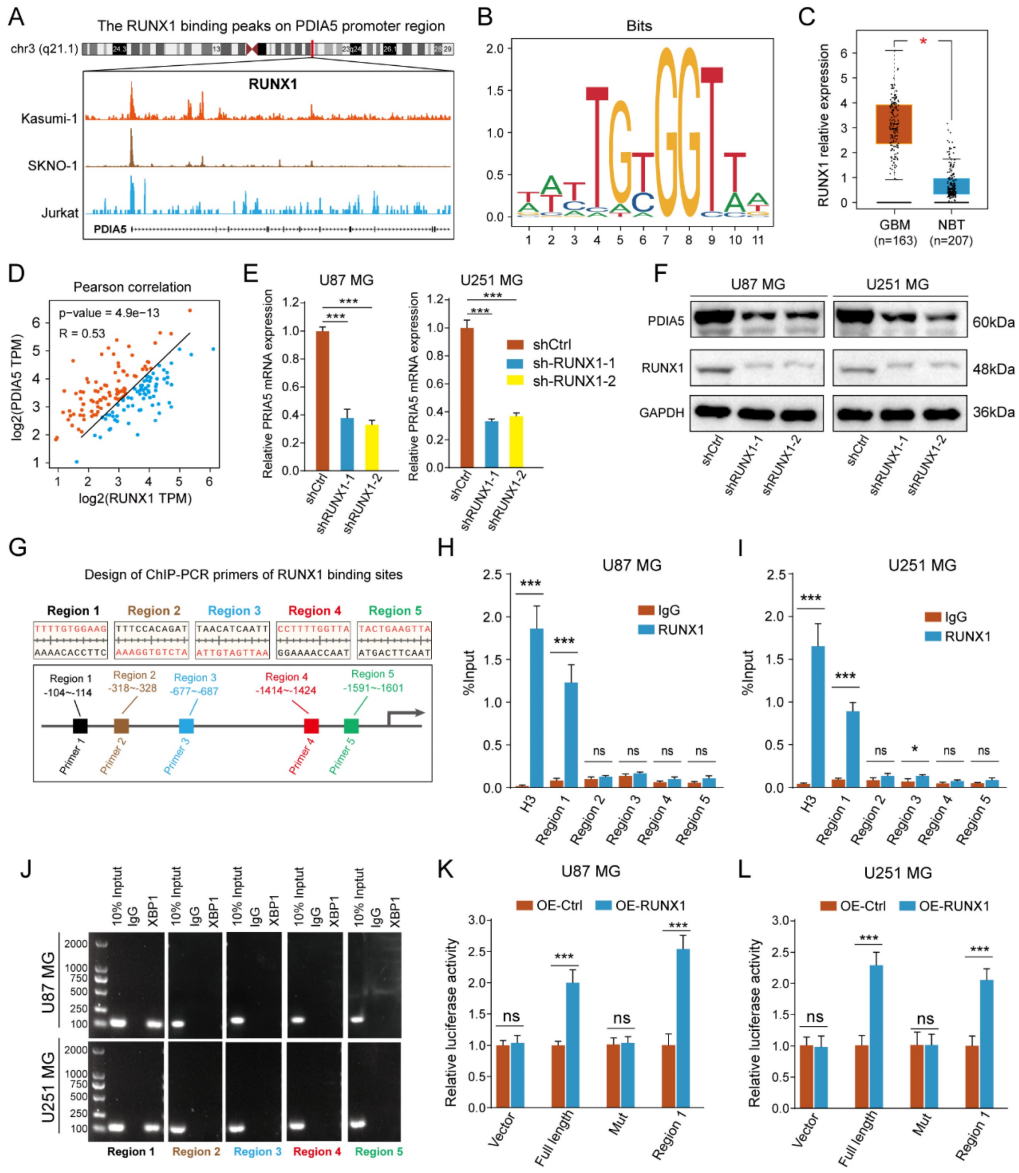
** (A)** Peak map of the binding of RUNX1 to the PDIA5 promoter region. **(B)** motif schematic diagram of transcription factor RUNX1 binding. **(C)** Differential expression of RUNX1 between GBM tissue and normal brain tissue. **(D)** Correlation analysis between RUNX1 and PDIA5. **(E)** The mRNA expression of PDIA5 decreased significantly in U87MG and U251MG cells after knocking down RUNX1 expression. **(F)** Protein expression of PDIA5 decreased significantly in U87MG and U251MG cells after knocking down RUNX1 expression.** (G)** Design of ChIP-PCR primers for transcription factor RUNX1 binding region. **(H, I)** Chain-PCR results showing the region 1 sequence (from approximately -104 to -114) on the PDIA5 promoter region was captured by RUNX1 protein immunoprecipitation in U87MG **(H)** and U251MG** (I)** cells. **(J)** DNA electrophoresis determines the amplification results of PCR products. **(K,L)** Dual luciferase reporter assays indicating the PDIA5 promoter binding region of RUNX1 in GBM cells.

To identify the transcriptional regulatory mechanism of PDIA5 expression in GBM cells, we used public websites and databases, including JASPAR and UCSC, to predict the upstream transcription factors of PDIA5. The binding peak of RUNX1 in the PDIA5 promoter region was the most remarkable **(Figure 8A)**. A motif for the binding of RUNX1 transcription factor was obtained from the JASPAR2022 database **(Figure 8B)**. Analysis on the GEPIA website showed that RUNX1 was significantly upregulated in GBM tissues compared to normal brain tissues **(Figure 8C)**, and its expression was significantly correlated with the expression of PDIA5 **(Figure 8D)**. Furthermore, we verified the regulation of RUNX1 on PDIA5 mRNA and protein expression by RT-qPCR and western blotting.

After knocking down RUNX1 protein expression, we found that the expressions of PDIA5 mRNA and protein were decreased **(Figure 8E-F)**. The prediction results of the RUNX1 and PDIA5 binding sites were obtained from the JASPAR 2022 database, and the top 5 sites were selected for verification in U87MG and U251MG cells **(Figure 8G)**. The transcription factor RUNX1 was also verified by ChIP-PCR to have the highest binding level in the predicted region 1 in both U87 and U251 GBM cells **(Figure 8H-I)**. Finally, the reliability of the ChIP-PCR results was further verified by DNA electrophoresis using the ChIP-PCR product from each region **(Figure 8J)**. Meanwhile, we performed a dual-luciferase reporter assay to verify direct binding of the RUNX1 transcription factor and the Region 1 sequence to the PDIA5 promoter. The full-length promoter sequence (-2000 to -1 bp) sequence of PDIA55, the clipped forms of the PDIA5 promoter including region 1 (-104 to -114 bp), and the full-length promoter sequence of PDIA5 with mutated region 1 were cloned into the vector plasmid, respectively. These results suggest that overexpression of RUNX1 activates the transcriptional activity of the PDIA5 promoter, but not that of the region 1 mutation sequence** (Figure 8K-L)**. In conclusion, these data indicated that region 1 of the PDIA5 promoter was the binding site of the RUNX1 transcriptional factor.

## Discussion

The cancer pathogenesis involves uncontrolled cell proliferation caused by abnormal expression and regulation of genes, which mainly involves the activation of proto-oncogenes and the loss of function of tumor suppressor genes 32. Normal cell proliferation requires the stimulation of mitotic growth signals, but in most cancer cells, the normal cell proliferation pathway is dysregulated to various degrees, and the dependence of tumor cells on exogenous growth stimulation is significantly reduced 33. In cancer tissues, proteins that can restrict cells to their original location are changed, and some intercellular adhesion factors interact with extracellular receptors to break homeostasis between cell junctions and the surrounding environments, leading to the promotion of cancer cell migration and invasion 34. The increases in energy demand reactive oxygen species (ROS) and protein synthesis, accelerate the occurrence of ER Stress, all of which require PDI proteins to maintain the correct disulfide bonding between cysteines, to keep protein homeostasis within tumor cells 35.

Although PDI family proteins were reported to be located primarily in the ER, but in our results we found that most of PDIA5 was located in the nucleus, and the immunofluorescence of PDIA5 in the Human Protein Atlas (HPA) dataset showed similar data. It was interesting that PDIA5 may not only function in the ER, which could explain how CCAR1, which is located primarily in the nucleus, can act as a substrate of PDIA5. PDIA5 is a potential target for cancer therapy 36. In the course of cancer therapy, PDIA5 can interact with RNASET2 to resist the killing ability of drugs on pancreatic cancer cells 37. Other studies have also shown that PDIA5 can regulate tumor progression by participating in multiple signaling pathways, such as the PI3K/AKT, the RTK, and the androgen receptor signaling pathways 38-40. Under ER stress, PDIA5 promotes the rearrangement of the disulfide bond of ATF6α to obtain an active conformation, thus affecting the progression of gliomas 41. Although the pro-oncogene role of PDIA5 in cancers has been reported previously, our study revealed another regulatory effect that PDIA5 plays in GBM cells, through the promotion of the expression of the protein CCAR1.

Cell cycle and apoptosis regulator 1 (CCAR1) is a regulator of apoptosis signaling and cell proliferation. Several chemical compounds that bind to CCAR1, effectively blocking its functions and promoting cell apoptosis, have been developed 42. In the process of chemotherapy-induced cancer cell apoptosis, CCAR1 is also one of the essential proteins involved 43,44. Furthermore, increasing research suggests that CCAR1 may play an important role in the malignant progression of cancers. In gastric cancer, the interaction between CCAR1 and β-catenin can promote the proliferation and migration of gastric cancer cells 45. In breast cancer, CCAR1 interacts with coactivators of estrogen receptor signaling to promote the proliferation of breast cancer cells 46,47. In lung cancer, CCAR1, as the target gene of miR-627-3p, affects the proliferation and invasion ability of lung cancer cells 48,49. In prostate cancer, CCAR1 depletion can inhibit the growth, migration, and invasion of prostate cancer cells, and reduce the tumorigenicity of prostate cancer cells *in vivo* 49. But how the CCAR1 protein is correctly formed and regulated in GBM cells is still unknown, our study revealed that PDIA5 promotes GBM cell malignancy by enhancing the expression of the CCAR1 protein, in a folding or maturation-facilitating manner.

According to existing reports, RUNX1 can regulate the progression of mesenchymal glioma by modulating the TGFβ pathway 50. In addition, RUNX1 promotes the malignant progression of GBM cells by regulating the JAK-STAT pathway 51 and regulates the migration, invasion, and angiogenesis of GBM cells through the p38/MAPK pathway 52. These studies indicate that RUNX1 is an oncogenic factor in GBM. In our investigation, we identified another regulatory pathway of RUNX1 in GBM, the RUNX1/PDIA5/CCAR1 pathway, and demonstrated its role in promoting the malignant progression of GBM both *in vitro* and *in vivo* by regulating the expression of PDIA5.

Some limitations also need to be addressed. Although we have demonstrated the oncogenic role of PDIA5 in GBM, clinical inhibition of PDIA5 in patients with GBM remains a significant challenge. Currently, no PDIA5-specific inhibitor is capable of penetrating the blood-brain barrier. Therefore, targeting PDIA5 through other biomedical engineering approaches, such as siRNA, antibodies, or specific gene-engineered drug delivery methods, may be more effective at this stage and could be very promising for the clinical therapy of patients with GBM.

## Conclusion

This study comprehensively analyzed and evaluated the role of PDIA5 in GBM both *in vitro* and *in vivo*, demonstrating the crucial functions of PDIA5 in proliferative and invasive regulation in GBM cells. Our findings support the importance of the RUNX1/PDIA5/CCAR1 signaling axis in GBM cell malignancy. We found that the expression of PDIA5 is not only upregulated in GBM tissues, but is also associated with a poor prognosis in patients with GBM. Our data indicate that PDIA5 plays an important role in the migration, invasion and proliferation of GBM cells. Rather than a direct regulation of GBM cell malignancy by PDIA5 protein, PDIA5 binds to the CCAR1 protein and regulates its expression by modulating protein folding or facilitating maturation via its -CXXC domain, thus controlling the malignant behaviors of GBM cells. Finally, we also proposed and provide evidence for transcriptional regulatory mechanism of PDIA5: The transcription factor RUNX1, which is highly upregulated in GBM samples and cells, can directly bind to the promoter region of PDIA5 and effectively regulate the expression of the PDIA5 protein. In general, the importance of the RUNX1/PDIA5/CCAR1 signaling axis in the progression of GBM was identified in this study, which is expected to be a potential target and a basic concept for the development of a new strategy to inhibit the malignant progression of GBM.

## Supplementary Material

Supplementary figures.

## Figures and Tables

**Figure 9:**
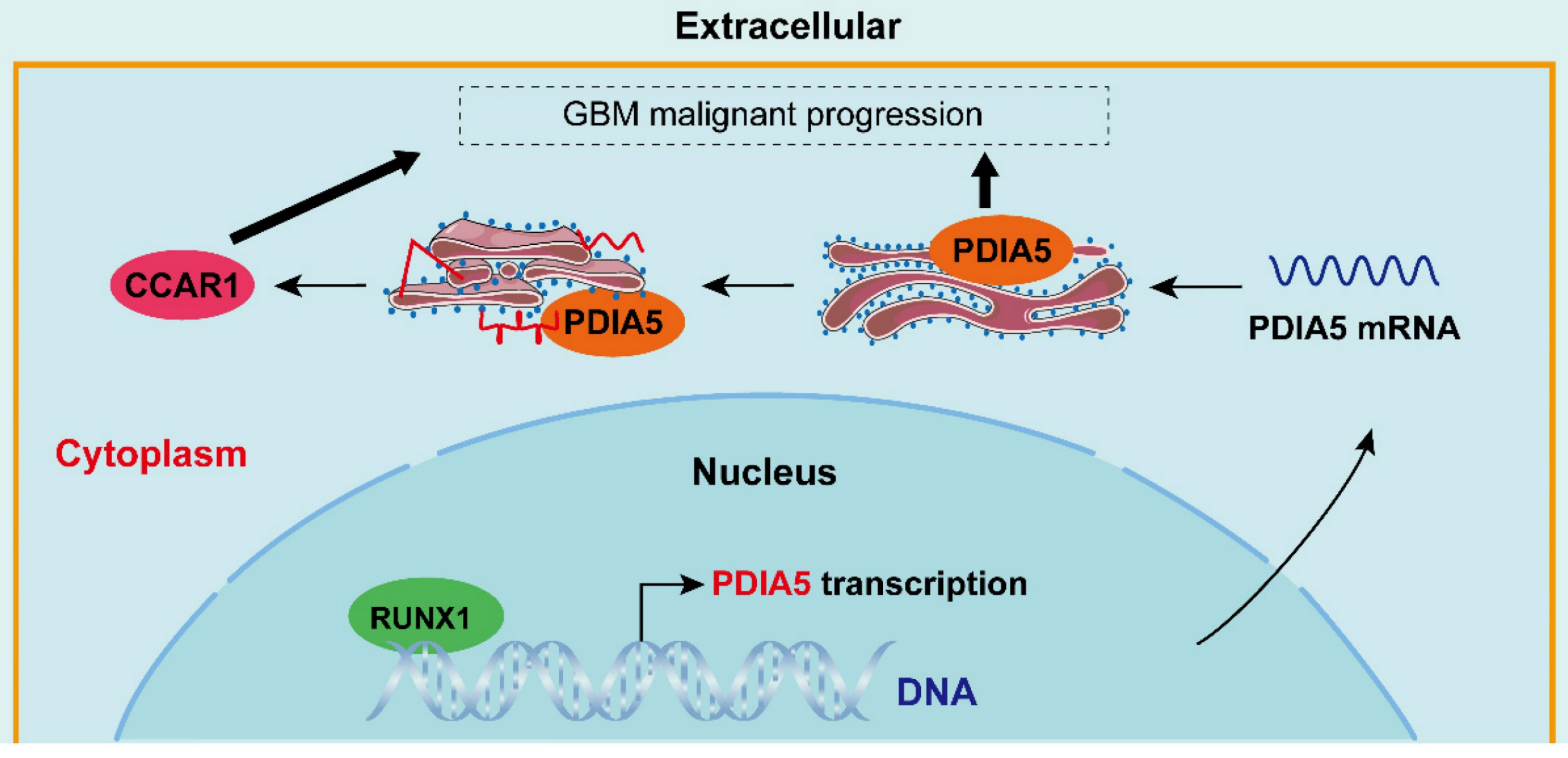
Simulated mechanism diagram of the RUNX1/PDIA5/CCAR1 axis in GBM. In GBM cells, PDIA5 is aberrant upregulated transcriptionally by the transcriptional factor RUNX1, which leads to higher PDIA5 protein expression. PDIA5 accelerates the cell division cycle and apoptosis regulator protein 1 (CCAR1) correct folding and maturation in an oxidoreductase manner, promoting the GBM cell proliferation and invasion.

## Abbreviations

GBM: glioblastoma multiforme
PDIA5: protein disulfide isomerase family A member 5
TCGA: The Cancer Genome Atlas
CGGA: Chinese Glioma Genome Atlas
GTEx: Genotype-Tissue Expression
ATCC: American Type Culture Collection
WB: Western Blot
IHC: Immunohistochemical staining
LC-MS: liquid chromatography mass spectrometry
ER: endoplasmic reticulum
ChIP: chromatin immunoprecipitation
shRNA: short hairpin ribonucleic acid
PCR: polymerase chain reaction
RPKM: Reads Per Kilobase per Million mapped reads
DEG: Differentially expressed gene
BBB: blood-brain barrier
