# Supplementary material for: RUNX1-PDIA5 Axis Promotes Malignant Progression of Glioblastoma by Regulating CCAR1 Protein Expression
Source: Int J Biol Sci. 2024 Aug 12;20(11):4364–81. doi: 10.7150/ijbs.92595 (PMC11379074; doi:10.7150/ijbs.92595)
Supplement: Supplementary file 1 — Supplementary figures. [file ijbsv20p4364s1.pdf]

Supplementary information

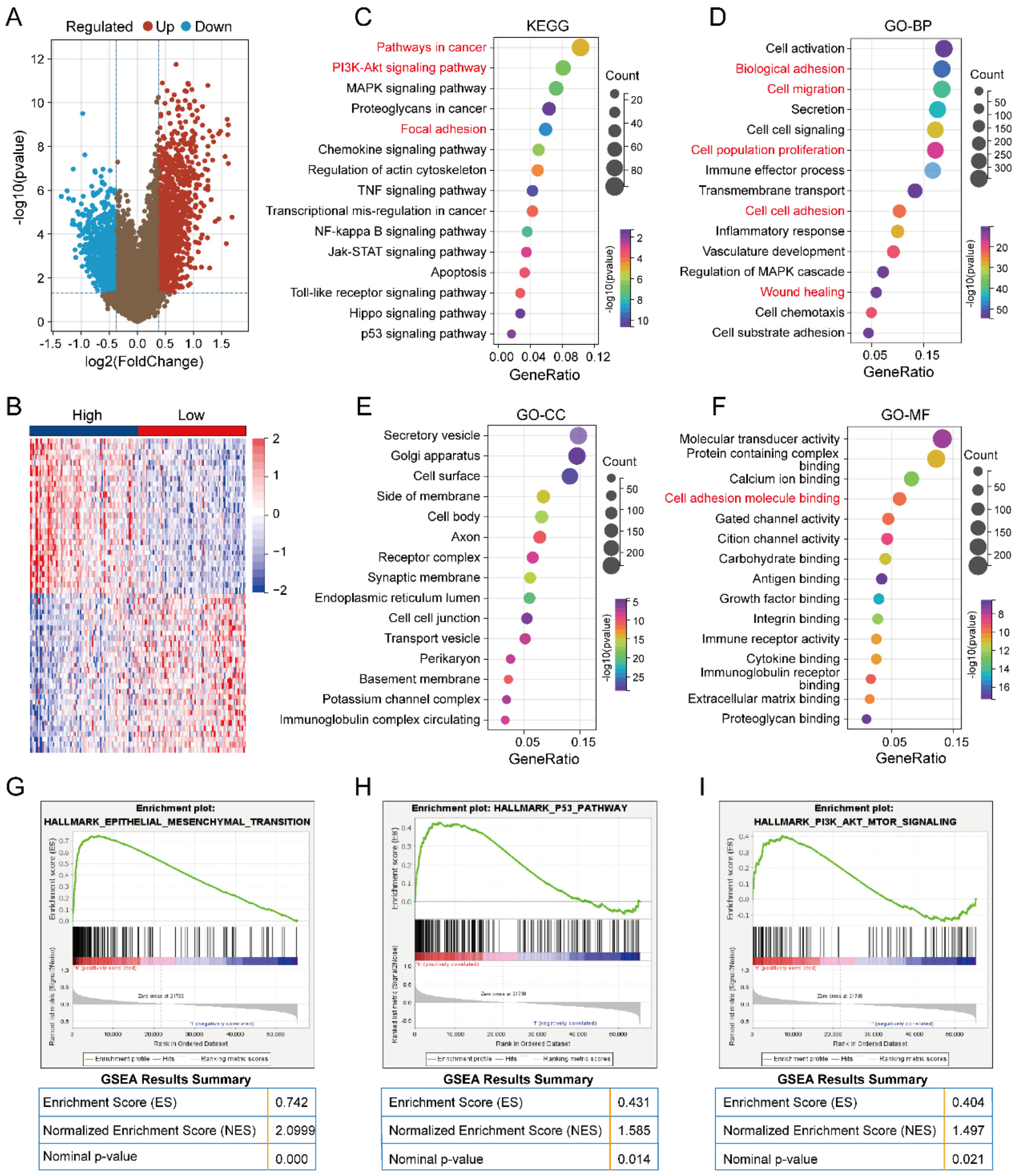

Figure S1. (A) Volcano map showing the distribution of differentially expressed genes between high and low PDIA5 expression in U251 GBM cells. CleRed represents positively related genes; Blue represents negatively correlated genes; Orange represents genes with a | multiple of | < 1.3. **(B)** Heat maps showing the clustering results of some differentially expressed genes between high-low expression groups. **(C)** Bubble diagram showing KEGG enrichment analysis results. **(D-F)** Bubble map showing GO

enrichment items (GO: Gene Ontology, gene function classification system; BP: Biological Process **(D)**; CC: Cell Component, cell component **(E)**; MF: Molecular Function **(F)**). Gene set associated with **(G)** Epithelial mesenchymal transition, **(H)** the "p53 pathway", and **(I)** "PI3K/AKT/MTOR signaling".

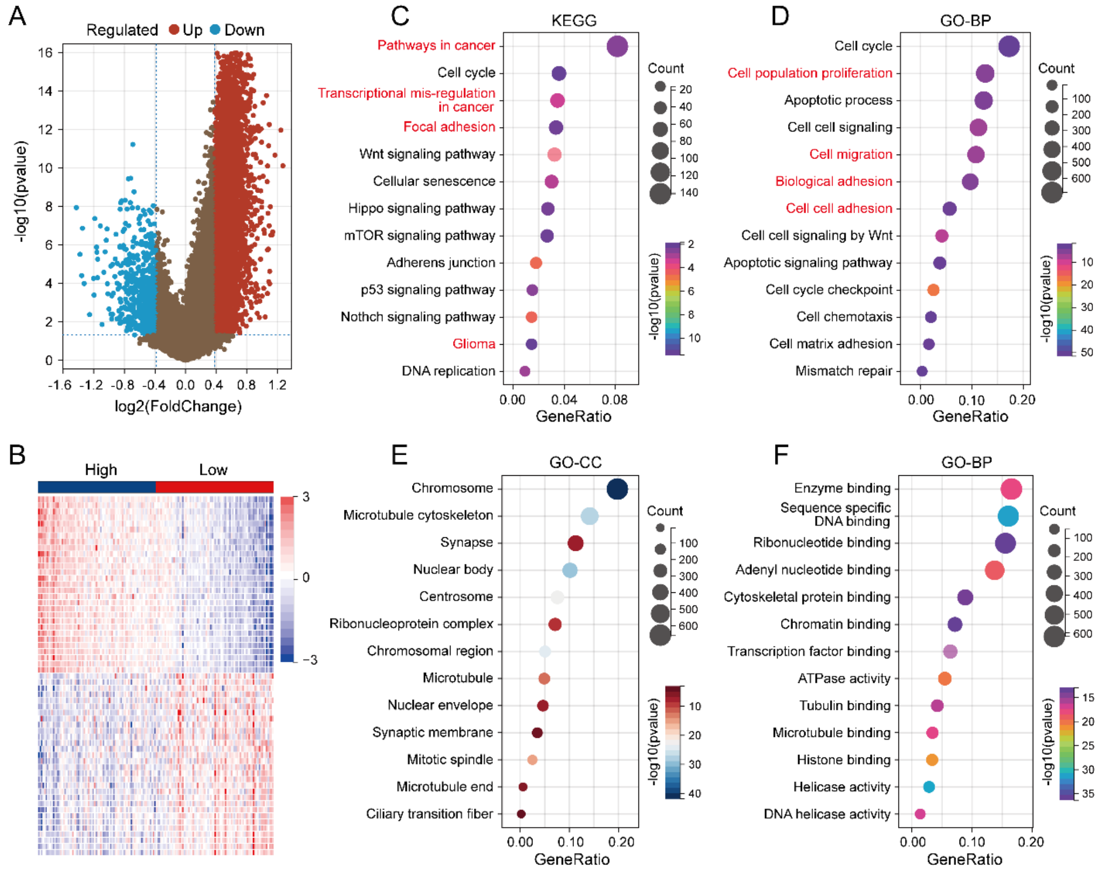

Figure S2. (A) Volcano map shows the distribution of differentially expressed genes between of GBM cells with high and low CCAR1 expression. Red represents positively related genes; Blue represents negatively correlated genes; Orange represents genes with a | log2(FoldChange)| < 1.3. **(B)** Heatmap showing the clustering results of differentially expressed genes between the high- and low-expression groups. **(C)** Bubble diagram shows the top KEGG pathway enriched terms. **(D-F)** Bubble plot showing the top GO enrichment items (GO: Gene Ontology, gene function classification system; BP: Biological Process **(D)**; CC: Cell Component **(E)**; MF: Molecular Function **(F)**).

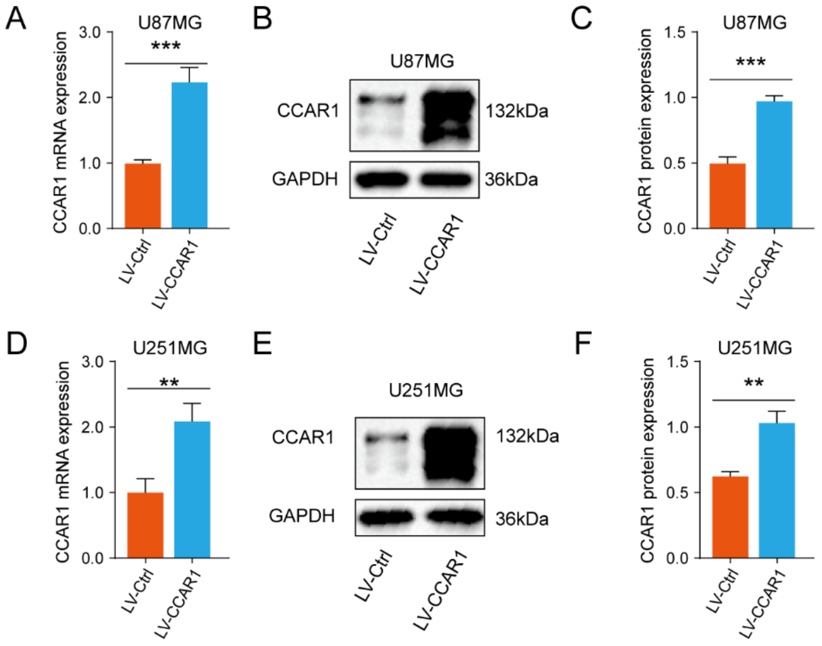

Figure S3. Effects of overexpression of CCAR1 in U87MG **(A-C)** and U251MG **(D-F)** cell lines as detected by RT-qPCR and WB.

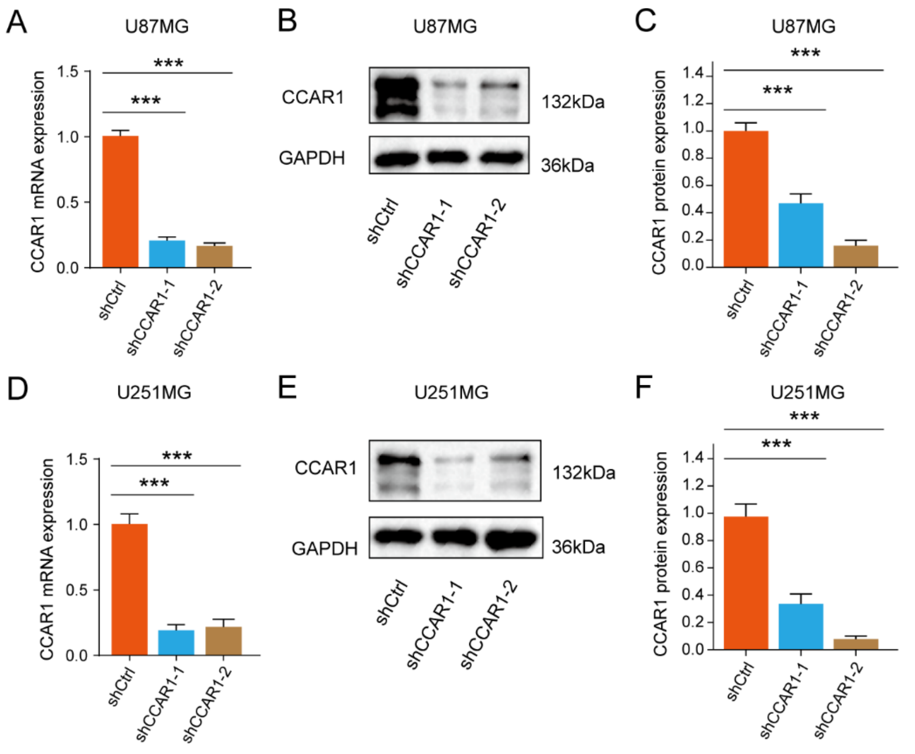

Figure S4. Effects of CCAR1 knock-down in U87MG **(A-C)** and U251MG **(D-F)** cell lines as detected by RT-qPCR and WB.

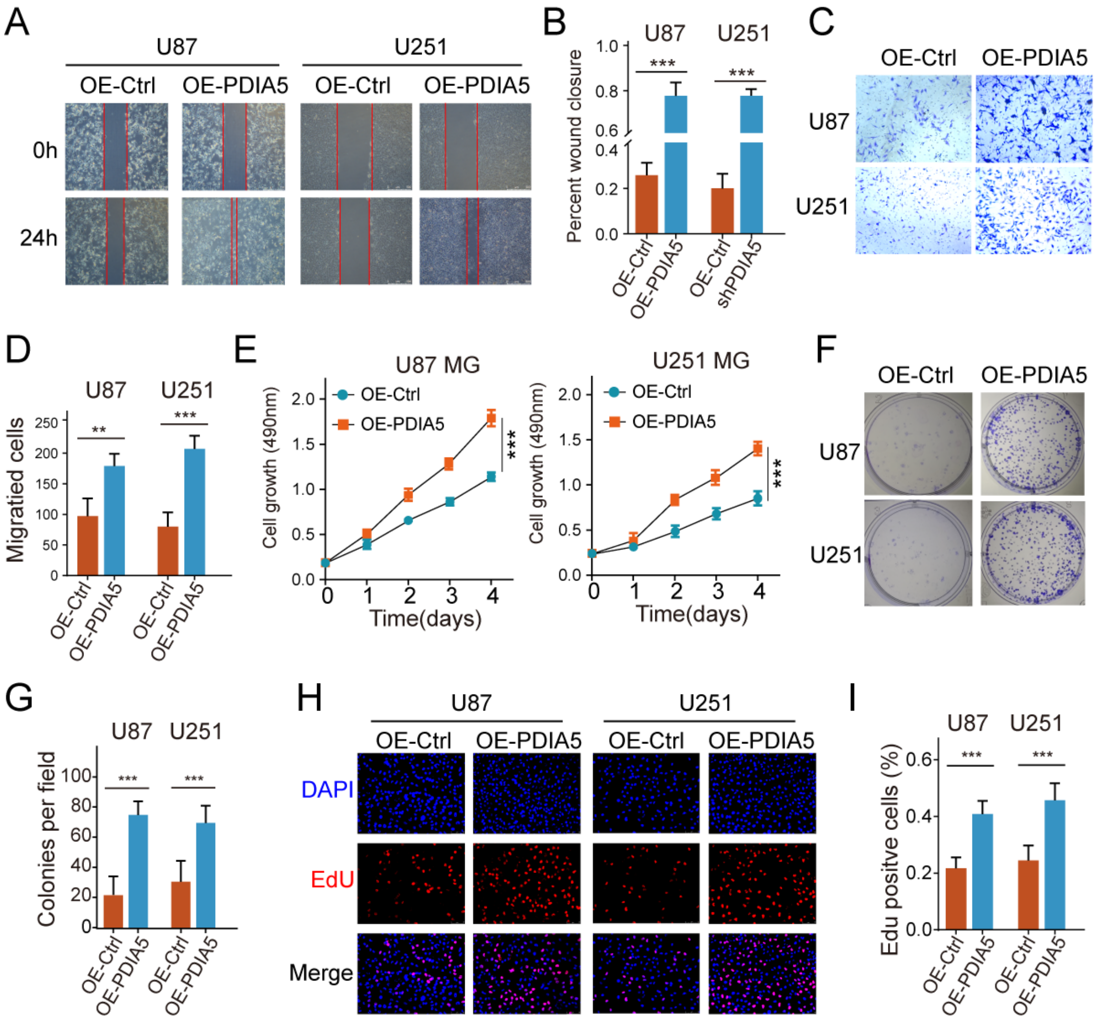

Figure S5. Effects of PDIA5 overexpression on wound-healing ability of **(A)** U87MG and **(B)** U251MG cells was detected by wound-healing experiment. Transwell invasion assay was used to detect the effects of PDIA5 overexpression on invasion ability of **(C)** U87MG and **(D)** U251MG cells. **(E)** CCK-8 assay, (**F-G**) colony formation assay, and (**H, I**) EdU assay verified the effects of PDIA5 overexpression on the proliferation ability of U87MG and U251MG cells.

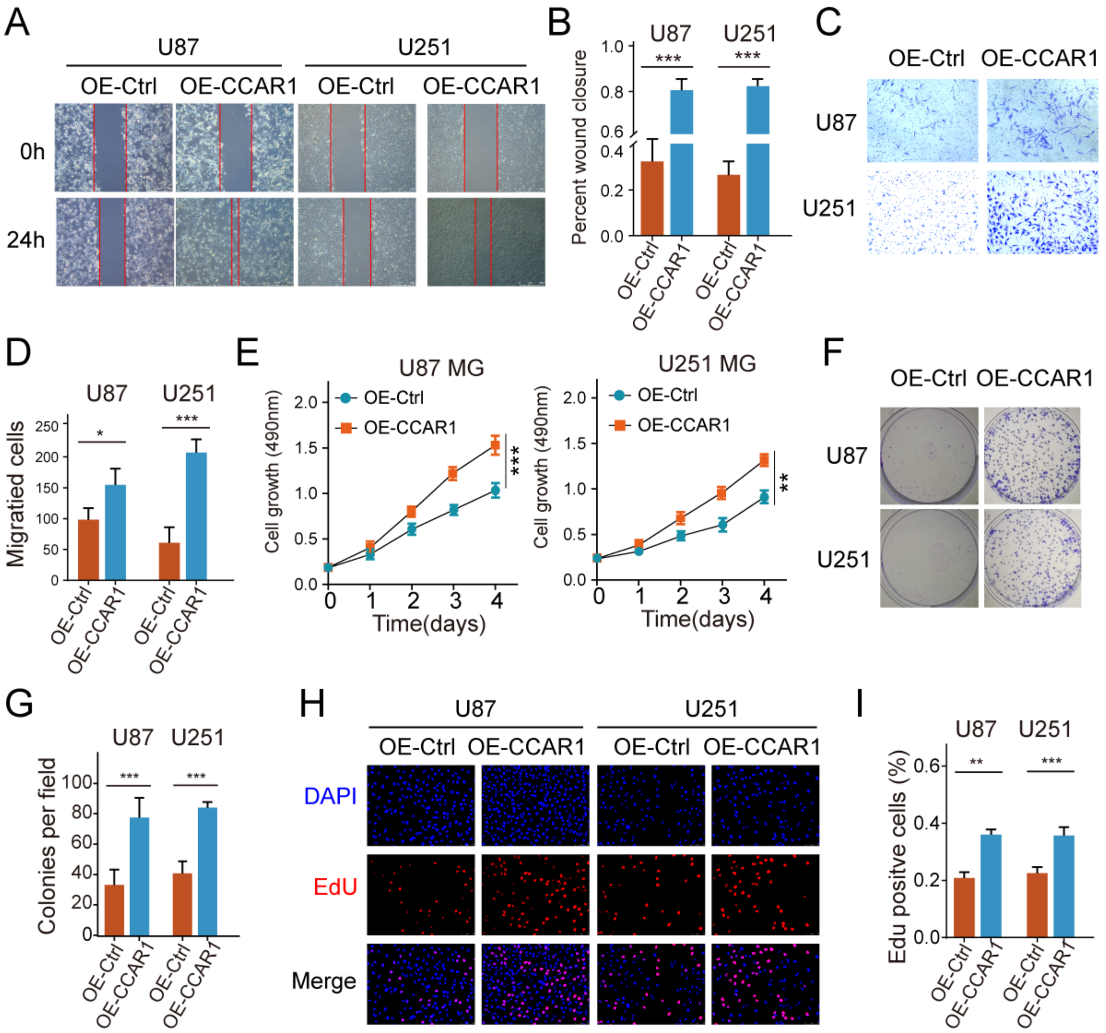

Figure S6. The effect of CCAR1 overexpression on wound-healing ability of **(A)** U87MG and **(B)** U251MG cells was detected by the wound-healing assay. Transwell invasion assay was used to detect the effects of CCAR1 overexpression on invasion ability of **(C)** U87MG and **(D)** U251MG cells. **(E)** CCK-8 assay, **(F, G)** Colony formation assay, and **(H, I)** EdU assay verified the effects of CCAR1 overexpression on the proliferation ability of U87MG and U251MG cells.
